# Tobacco Use and Exposure to Second-Hand Smoke among Urban Residents: A Community-Based Investigation

**DOI:** 10.3390/ijerph120809799

**Published:** 2015-08-18

**Authors:** Zhaorui Xu, Hongzhi Han, Cheng Zhuang, Chunyu Zhang, Ping Zhao, Yan Yao

**Affiliations:** 1 Changchun Health Education Centre, Changchun, Jilin 130021, China; E-Mails: arui1103@126.com (Z.X.); zcy20150701@sina.com (C.Z.); zhaoping19@163.com (P.Z.); 2 Editorial Office of Journal of Jilin University Medicine Edition, Changchun, Jilin 130012, China; E-Mail: hanhz@jlu.edu.cn; 3 Changchun Institute of Health Supervision, Changchun, Jilin 130021, China; E-Mail: zhuangcheng@hotmail.com; 4 Department of Epidemiology and Biostatistics, School of Public Health, Jilin University, Jilin 130021, China

**Keywords:** tobacco, second-hand smoke

## Abstract

*Objectives*: In 2005, China acceded to the World Health Organization Framework Convention on Tobacco Control (WHO FCTC), the foundation for the global fight against tobacco. Certain cities in China have established local regulations to control tobacco use ahead of national policy; however, without the enforcement of statutory law, some of these regulations are merely lip service. The aim of the study was to assess the effects of city policy on smoking prevalence and on second-hand smoke (SHS) exposure status among non-smokers in Changchun City. *Methods*: A cross-sectional survey covering a multiple-stage, representative sample of the urban population aged ≥15 years was conducted between 1 Dec 2013 and 31 Jan 2014. The WHO and the US Centers for Disease Control and Prevention developed the questionnaires used, which included demographic characteristics, smoking behaviors and SHS exposure status. *Results*: Overall cigarette smoking prevalence was 23.5%; daily cigarette smoking prevalence was 21.2%. Smoking prevalence and cigarettes consumed per day was higher among men (*p* < 0.05) and those aged 45–64 years (*p* < 0.05). Among current smokers, 8.1% planned to quit within 12 months; 53.4% had no intention of quitting. Overall SHS exposure prevalence was 41.9% (workplace) and 34.1% (at home) over the previous 30 days. The weighted workplace SHS exposure prevalence increased with age. *Conclusions*: The high proportion of smokers with no intention of quitting and the high level of SHS exposure may constitute one of the most significant barriers to successful smoking cessation in the city. A continued drive to promote full implementation of the WHO FCTC is still needed.

## 1. Introduction

Smoking is one the leading causes of preventable death in the world [1]. It is associated with cancer, coronary heart disease, respiratory disease and stroke [2,3]. The smoking behavior not only harms the health of the smokers but also indirectly harms the health of the people around. Second-hand smoke (SHS)—defined as the mixture of smoke from the burning end of a cigarette, cigar or pipe and the exhaled fumes of the smoker—is at least as dangerous as smoke from smoking cigarettes directly. There are over 4000 chemicals in cigarette smoke, and SHS actually contains higher amounts of harmful chemicals, such as nicotine, tar, carbon monoxide, ammonia and heavy metals, compared with what is inhaled by the smoker. It has been reported that the number of deaths caused by tobacco has now reached 6 million, which exceeds the combined death tolls of AIDS, tuberculosis and malaria in the world [4]. Researchers predict that this figure will increase to 8 million by 2030, and most of these deaths are likely to be largely concentrated in the developing world [5].

Tobacco consumption has a long history in China. According to a report from the Global Adult Tobacco Survey (GATS) 2010 program, at that time there were more than 300 billion smokers in China and 740 billion non-smokers exposed to SHS [6]. Since 2000, about a million people have died from smoking-related diseases in China, and of these, more than 100 thousand people have died from disease caused by exposure to SHS. If this rate continues, this figure will have increased to 2 million by 2030, and to 3 million by 2050 [7]. As tobacco smoke has been acknowledged as one of the main causes of indoor air pollution in China, and with increasing awareness of the dangers of passive smoking to the public, [8] a call to limit tobacco production and prohibit smoking in public places is now gaining momentum.

Globally, up to 2012, more than 100 countries have formulated a ban on smoking in public places, and of these, 44 countries have implemented a complete smoking ban. China joined the World Health Organization (WHO) Framework Convention on Tobacco Control (WHO FCTC) in 2005, which came into force in 2006 [9]. To date, however, no national law has been implemented in China, although certain cities have established some local regulations or launched a cessation program to control tobacco use ahead of national policy. However, without the enforcement of statutory law, some of these regulations merely pay lip service to the problem. Changchun City is one such city that has implemented a local ban. As capital of Jilin Province in China, Changchun established a partial smoke-free legislation in 1996 and engaged in a smoke-free city program funded by the Gates Foundation in 2010. Throughout this period, the local government has been launching efforts to deal with the significant challenge of controlling tobacco use. However, what is the situation with regard to smoking in Changchun now? Is smoking less prevalent now? To the best of our knowledge, no studies have evaluated this issue in Changchun using a representative population sample of adults. In our study, we performed a cross-sectional survey by a three-stage sampling strategy to explore participants' smoking behaviors and exposure to SHS in Changchun. The aims of this study were to assess the prevalence of smoking and to understand SHS exposure status (level and location of exposure) among the general population following the drive to discourage smoking.

## 2. Methods

A cross-sectional survey assessing tobacco consumption and exposure to SHS was carried out in a representative sample of the urban population aged 15 years and over from 1 Dec 2013 to 31 Jan 2014 in Changchun, Jilin Province, China. Changchun has five main urban areas in the city: Chaoyang District, Nanguan District, Erdao District, Kuanchen District and Lvyuan District. The study participants were selected by means of a three-stage sampling strategy with stratification in the first-stage units, *i.e*., communities. To guarantee representativeness, communities were stratified by geographical region and the size of the district. Second-stage units were households in the previously selected communities. A sample of the required number of households was selected according to the population size of the villages using probability sampling. Third-stage units were residents in the previously selected households, where only one eligible person was selected at random. The inclusion criteria were that subjects should be: (1) permanent residents living within one of the five main urban districts in Changchun at least one year before the start of the survey; (2) aged ≥15 years; and (3) willing to participant voluntarily in the survey after being informed. Those who lived in a dormitory, hospital, barrack, nursing home or prison were excluded from the study. The population sample size for the survey was calculated to be 2000. In total, 2,213 residents provided informed consent for their participation, and were interviewed. For participants aged 15 years, informed parental consent was sought and granted. The response rate was 99.1%.

This study formed part of the Chinese Tobacco Questions for Survey (TQS) 2013 program. Questionnaires used in the program were developed by the WHO and the US Centers for Disease Control and Prevention (US CDC), and it was the core subset of questionnaires used in GATS. In order to accommodate the tobacco-use epidemic in China, Chinese Center for Disease Control and Prevention (China CDC) made some adjustments to the questionnaire. The contents of the TQS mainly included demographic characteristic, smoking behaviors and exposure to SHS. A current smoker was defined as a person who was smoking, whether daily or not, at the time of the survey. A former smoker was defined as a person who had smoked more than 6 months before but who had stopped 1 week before being recruited for the survey, and a non-smoker as a person who reported to have never smoked. Non-smokers exposed to SHS reported that they had been exposed to SHS at home or in the workplace over the previous 30 days. Under the guidance and support of the China CDC and US CDC, Changchun Health Education Authority completed the investigation. During the survey, quality assurance measures were applied to the process of sampling, training of the data collectors, data collecting and data management. The results in our study could be used to compare with those of other TQS surveys.

To obtain a representative set of population data, all responses were population weighted by age and sex. Prevalence rates and 95% confidence intervals (CIs) were weighted by age and sex to population characteristics using Taylor's series method [10]. Population data were obtained from the 2010 Jilin census. The prevalence of tobacco consumption with 95% CIs was estimated overall and by sex, age group, and level of education. Differences by age, by sex and by educational level were tested using the Rao-Scott χ^2^ test or by variance analysis adjusted for survey design and weight. Significance was determined as *p* < 0.05, using a 2-tailed test. All statistical analyses were performed using SPSS version 19.0 (SPSS Institute Inc.).

## 3. Results

Data on the prevalence of cigarette smoking were based on the responses of 1178 women and 1026 men. As shown in Table 1, the overall prevalence of cigarette smoking was 23.5%–43.2% in males and 3.8% in females. The overall prevalence of daily cigarette smoking was 21.2%–39.4% in males and 3.1% in females.

**Table 1:** Prevalence of tobacco consumption among residents in Changchun, weighted prevalence (95%CI).

Tobacco Consumption	Overall	Men	Women
*Current smoker*	23.5 (20.6–26.7)	43.2 (38.6–47.9)	3.8 (2.5–5.6)
Every day smoker	21.2 (18.6–24.1)	39.4 (34.8–44.1)	3.1 (2.1–4.6)
Occasionally smoker	2.2 (1.3–3.8)	3.8 (2.1–6.7)	0.7 (0.3–1.5)
*Former smoker*	3.8 (2.9–7.1)	6.5 (3.9–10.5)	1.1 (0.5–3.2)
Every day smoker ever but not currently	2.4 (1.5–3.7)	3.9 (2.5–6.1)	0.8 (0.4–1.9)
Occasionally smoker ever but not currently	1.3 (0.7–2.3)	2.3 (1.3–4.1)	0.2 (0.0–1.1)
*Non-smoker*	72.9 (69.1–76.3)	50.6 (45.3–55.8)	95.2 (92.8–96.8)

The prevalence of smokers was higher among men (*p* < 0.05) and among those aged 45–64 years (*p* < 0.05). Among those aged less than 65 years, the prevalence of cigarette smoking increased from 15.2% in the 15- to 24-year age group to 26.9% in the 45- to 64-year age group (Table 2). The weighted mean number of cigarettes per day (CPD) per person among all adult daily smokers in Changchun was 14.9 cigarettes. Men consumed more cigarettes than did women per day (15.3 ± 8.4 *vs*. 10.7 ± 6.5; t = 3.248, *p* < 0.05). By age, daily smokers aged 45–64 years consumed the most CPD (16.2 ± 9.3), followed by smokers aged 25–44 years (15.5 ± 7.8), smokers aged 65 years and over (11.1 ± 5.9), and smokers aged 15–24 years (10.0 ± 5.4). No significant differences were found in the number of CPD by level of education (*p* > 0.05).

**Table 2:** Prevalence of tobacco consumption among current smokers by sex, age group and level of education.

Characteristic	N	% Overall	N of Smokers	Smoking Prevalence (%)	*χ^2^*	*p*
*Sex*					507.131	<0.001
Men	1,026	50.0	462	43.2		
Women	1,187	50.0	53	3.8		
*Age (year)*					9.428	<0.001
15–24	206	15.7	33	15.2		
25–44	967	39.1	237	26.2		
45–64	759	32.2	200	26.9		
≥65	281	13.0	45	17.0		
*Level of education*					2.638	>0.05
Primary or less	200	9.9	50	25.8		
Junior middle school	490	22.9	116	23.1		
Senior middle school	680	33.8	190	25.8		
College or more	623	33.4	156	25.6		

Table 3 shows the distribution of smoking cessation behaviors by sex, age group and level of education. Over the previous 12 months, 24.5% of smokers had tried to quit, and more women than men had tried to quit (*p* < 0.05). Plans to quit were more prevalent among the younger age group (<25 years); specifically, higher proportions of younger respondents planned to quit within 12 months or were planning to quit within more than 12 months. Overall, only 8.1% of smokers planned to quit smoking within 12 months, and of these, more than half (53.4%) had no intention of quitting.

**Table 3:** Prevalence of smoking cessation among current smokers by sex, age group and level of educations in the last 12 months, weighted prevalence (95%CI).

Characteristics	Smoking Quitting Behavior			
	Have Tried to Quit Smoking In The Last 12 Months	Plan to Quit Smoking Within 12 Months	Plan to Quit Smoking, But Not Within 12month	Have No Desire to Quit Smoking
*Overall*	24.5 (18.4–31.8)	8.1 (4.9–13.2)	30.9 (22.4–40.9)	53.4 (43.4–63.1)
*Sex*				
Men	23.2 (17.3–30.4)	7.6 (4.4–12.9)	31.3 (22.2–42.0)	53.5 (42.5–64.1)
Women	39.2 (22.7–58.7)	14.2 (7.1–26.4)	25.9 (16.0–39.1)	52.8 (36.4–68.6)
*Age(years)*				
15–24	25.1 (13.4–42.1)	14.3 (7.6–25.3)	43.7 (31.5–56.7)	34.2 (21.8–49.3)
25–44	19.4 (13.3–27.3)	4.7 (2.4–8.9)	33.4 (23.2–45.4)	55.7 (43.6–67.3)
45–64	30.3 (20.9–41.7)	10.4 (5.8–17.8)	27.8 (18.3–39.9)	52.2 (40.1–63.9)
≥65	24.6 (13.9–39.7)	8.7 (2.4–26.9)	17.6 (8.5–32.9)	67.4 (48.5–82.0)
*Level of education*				
Primary or less	27.8 (16.9–42.1)	13.5 (5.8–27.9)	13.8 (6.4–27.1)	66.0 (52.3–77.5)
Junior middle school	27.2 (16.7–41.0)	7.8 (3.4–16.9)	30.3 (19.4–43.8)	52.6 (40.2–64.8)
Senior middle school	26.7 (18.7–36.5)	6.9 (2.6–17.1)	33.0 (21.6–46.9)	54.7 (40.1–68.5)
College or more	19.7 (10.7–33.6)	6.1 (2.4–14.7)	30.4 (21.0–41.9)	54.4 (41.0–67.2)

As shown in Table 4, among non-smokers, the overall prevalence of exposure to SHS was 41.9% in the workplace, and 34.1% at home, over the previous 30 days. More men than women were exposed to SHS in the workplace (*p* < 0.05), while on the contrary, more women than men were exposed to SHS at home (*p* < 0.05). The weighted prevalence of exposure to SHS in the workplace increased with age, while non-smokers aged 24 years or less were more often exposed to SHS at home. No significant differences were found in the weighted prevalence of exposure to SHS in the workplace by level of education (*p* > 0.05). The prevalence of exposure to SHS at home was higher among those with secondary education only (*p* < 0.05), and lower (*p* < 0.05) among those with a higher educational level.

**Table 4:** Prevalence of second-hand smoke (SHS) exposure among non-smokers by sex, age group and level of education in the last 30 days.

Characteristics	At Workplace				At Home			
	N	N of SHS Exposure	Weighted Percentage (%)	*Χ^2^*	N	N of SHS Exposure	Weighted Percentage (%)	*Χ^2^*
*Overall*	873	386	41.9		1613	520	34.1	
*Sex*				8.941 ^b^				51.107 ^a^
Men	323	164	47.6		534	109	22.8	
Women	550	222	38.1		1079	411	40.8	
*Age(years)*				5.972 ^b^				7.944 ^c^
15–24	104	38	33.5		164	65	43.6	
25–44	557	241	41.5		686	210	30.9	
45–64	206	104	49.8		539	183	34.7	
≥65	7	-	-		224	62	29.7	
*Level of educations*				0.512 ^c^				19.799 ^a^
Primary or less	14	6	-		147	53	37.5	
Junior middle school	120	51	38.6		360	140	39.9	
Senior middle school	256	118	45.0		479	146	34.5	
College or more	378	173	44.6		453	113	22.6	

Notes: ^a^ *p*<0.001, ^b^ *p*<0.05, ^c^ *p*>0.05; - No result could show if unweighted figures less than 25.

## 4. Discussion

This study showed a high prevalence of smokers in Changchun of China, where 23.5% of the adult population smoked, and 21.2% smoked every day. Although the overall smoking prevalence was lower than that revealed by the Chinese national data (27.3%), [11] and lower than the average results for adults of Jilin Province (31.8%), [12] it was still higher than that reported in one Spanish study (20.7%) [13] and one US study (18%) [14]. Our study indicated a slightly lower level of smoking among the urban residents of Changchun City as compared to provincial and national level [11,12], indicating improvement of smoking behaviors and that the city policy is effective. Measures of partial smoking-free policy, pictorial health warnings, and educational campaigns to control smoking and use of tobacco may have increased the momentum towards tobacco control in the city and may thus have contributed to the slight reduction observed in the prevalence of smoking as compared to provincial and national level [11,12]. However, in terms of smoking cessation, more than half of the current smokers (53.4%) in Changchun had no intention of quitting. This proportion was slightly higher than that reported in a study involving national data in 2010 (45%) [9]. Our study revealed conflicting information regarding tobacco control in Changchun. It is worthy of consideration that the high proportion of refractory smokers may constitute one of the most significant barriers to successful smoking cessation in the city. Nowadays, there is not so much nicotine-based smoking-cessation clinics in Changchun City. In order to reduce smoking prevalence of Changchun City, interventions such as building more such clinics and designing preferential policies to help those quit smoking could be utilized in the future. A continued drive to promote full implementation of the WHO FCTC is still needed.

In the present study, the percentage prevalences of overall smoking and daily smoking among men were 43.2% and 39.4% respectively, which were both higher than those found among women (3.8% and 3.1% respectively). In addition, the consumption of CPD was also higher in men than in women (15.3 ± 8.4 *vs.* 10.7 ± 6.5). The data indicate that men should be the key target in the efforts to control smoking in the urban areas of Changchun. Our results are in line with those of a study carried out in 9 provinces of China for the general population in a similar period [15]. In our study, it is promising that the prevalence of smoking among women in Changchun is lower than that among men, is less than the average rate among women in Jilin Province, [12] and has never been higher than in most western countries [16,17]. However, we have found that many women are exposed to SHS, which is as dangerous as smoking cigarettes directly. In the present study, 95.2% of women were non-smokers. Of these, 38.1% were exposed to second-hand cigarette smoke in the workplace and 40.8% at home. To sum up, at least 40% of women in Changchun were at risk of the harmful effects of tobacco. Furthermore, the evidence showed an upward trend of smoking prevalence among women in China, especially among the young, *i.e*., those aged less than 18 years [18]. In this sense, the sex gap is narrowing. It has been proved that smoking is the single most common cause of cancer worldwide, [19] being associated with colorectal, head and neck, lung, cervical, ovarian, and stomach cancer [20]. Thus, reducing the health risk from tobacco among women not only concerns efforts to reduce the prevalence of smoking, but more importantly, efforts to reduce their chances of being exposed to SHS. However, achieving this goal may rely heavily on legislation to reinforce a more conducive social environment, taking account of all walks of life and raising public awareness of tobacco control.

Those aged 45–64 years had the highest prevalence of cigarette smoking and consumed the most CPD when compared with other age groups. These results are consistent with those of previous studies investigating the relationship between age and nicotine dependence [21,22]. Most participants continued to smoke because of a nicotine addiction. Previous studies indicated that smoking behavior and nicotine dependence typically progress from the early 20s through to middle age [21]. With increasing age, the nature of the relationship between nicotine dependence and age changes. This may be on account of the higher level of nicotine binding affinity and nicotine metabolism reported in older adults compared with younger adults, in whom a reduced addictive potential of nicotine-related substances has been observed [23,24].

The main strengths of this study are its large sample size and its population representativeness. It is also noteworthy that the Changchun Tobacco Control Measures have been ratified by the Changchun authorities since 1 Mar 2014, which, so far, constitutes the strictest smoking ban in public spaces and is aimed at protecting individuals from SHS. The results of our study could be viewed as the baseline data from which to explore the effectiveness of new policies in the future. To our knowledge, this is the first study to assess the impact of the Changchun formal smoke-free legislation on the prevalence of smoking. Based on our findings, policy-makers should consider our observations when designing tobacco-control programs to ensure the effectiveness of their policies in Changchun.

There are several limitations to our study. First, it was a cross-sectional study, which limited our capacity to explore causal relationships. Second, self-reported data were used, which may be influenced by social desirability and bias. Third, the reasons for not quitting smoking were not explored. Previous studies reported that subjects' attitudes to quitting may be influenced by educational campaigns, smoking bans, sex, education level, population density, household income, and number of CPD [1,25]. These factors will be examined in future studies.

## 5. Conclusions

This study examined the effects of policy in terms of prevalence of smoking and SHS exposure status (level and location of exposure), among the residents of urban areas in Changchun. The results showed that there are two main features of tobacco control in Changchun: (1) a slightly lower prevalence of smoking than that at a national level, but a higher proportion of individuals with no intention of quitting; (2) a lower prevalence of smoking in women than in men, but a high degree of exposure to SHS both in the workplace and at home for men and women respectively. It is easy to ignore the low prevalence of smoking cessation and high prevalence of exposure to SHS when evaluating the effectiveness of tobacco-control policy. Even though this is a multifactorial area of investigation, it is vital that we explore the significance of these findings in more depth.
